# L1CAM, CA9, KLK6, HPN, and ALDH1A1 as Potential Serum Markers in Primary and Metastatic Colorectal Cancer Screening

**DOI:** 10.3390/diagnostics10070444

**Published:** 2020-06-30

**Authors:** Francis Yew Fu Tieng, Nadiah Abu, Surani Sukor, Zairul Azwan Mohd Azman, Norshahidah Mahamad Nadzir, Learn-Han Lee, Nurul Syakima Ab Mutalib

**Affiliations:** 1 UKM Medical Molecular Biology Institute (UMBI), Universiti Kebangsaan Malaysia, Cheras, Kuala Lumpur 56000, Malaysia; p103431@siswa.ukm.edu.my (F.Y.F.T.); nadiah.abu@ppukm.ukm.edu.my (N.A.); shedanazir@ppukm.ukm.edu.my (N.M.N.); 2 Prima Nexus Sdn. Bhd., Kuala Lumpur 50470, Malaysia; surani@primanexus.com.my; 3 Colorectal Unit, Department of Surgery, Faculty of Medicine, Universiti Kebangsaan Malaysia Medical Centre, Kuala Lumpur 56000, Malaysia; zairulazwan@ppukm.ukm.edu.my; 4 Novel Bacteria and Drug Discovery Research Group, Microbiome and Bioresource Research Strength, Jeffrey Cheah School of Medicine and Health Sciences, Monash University Malaysia, Subang Jaya 47500, Malaysia

**Keywords:** serum biomarkers, screening, colorectal cancer, metastasis, chemotherapy, noninvasive

## Abstract

Background: Colorectal cancer (CRC) screening at the earlier stages could effectively decrease CRC-related mortality and incidence; however, accurate screening strategies are still lacking. Considerable interest has been generated in the detection of less invasive tests requiring a small sample volume with the potential to detect several cancer biomarkers simultaneously. Due to this, the ELISA-based method was undertaken in this study. Methods: Concentrations of neural cell adhesion molecule L1 (L1CAM), carbonic anhydrase IX (CA9), mesothelin (MSLN), midkine (MDK), hepsin (HPN), kallikrein 6 (KLK6), transglutaminase 2 (TGM2) aldehyde dehydrogenase 1 family, member A1 (ALDH1A1), epithelial cell adhesion molecule (EpCAM), and cluster of differentiation 44 (CD44) from blood serum of 36 primary CRC and 24 metastatic CRC (mCRC) were calculated via MAGPIX^®^ System (Luminex Corporation, USA). Results: Significantly increased concentration (*p* < 0.05) of three serum biomarkers (L1CAM, CA9, and HPN) were shown in mCRC when compared with primary CRC. HPN and KLK6 showed significant differences (*p* < 0.05) in concentration among different stages of CRC. In contrast, levels of HPN and ALDH1A1 were significantly elevated (*p* < 0.05) in chemotherapy-treated CRC patients as compared with nontreated ones. Conclusion: Serum biomarkers could act as a potential early CRC diagnostics test, but further additional testings are needed.

## 1. Introduction

Colorectal cancer (CRC) is one of the most prevalent diseases with an alarming increase in incidence and mortality, particularly in developing countries [1]. According to Bray et al., in 2018, CRC was the third most prevalent diagnosed cancer and placed second for all cancer-related deaths [2]. Approximately 30% to 50% of the newly diagnosed CRC patients will quickly progress into later stages/metastatic CRC (mCRC), and their 5 year survival rate was around 50% to 60% [3,4]. It is beyond doubt that early detection, notably when the cancer lesions are localized and easy to remove, reduces the mortality and incidence of CRC [5]. Over the years, various methods were proposed as screening tools for CRC in an attempt to decrease their high mortality rate [6].

Up to date, the most accurate screening test available for CRC is colonoscopy. Colonoscopy is highly sensitive and specific as it enables examination of the complete colon and allows the removal of precancerous polyps [7,8]. However, its low compliance rate due to the invasive nature, dietary restriction and extensive bowel preparation requirement, frequent repeating measure (every 3 to 5 years), risk of perforation (1 in 1000 and 10,000 colonoscopies), unstandardized colonoscopy procedures and histopathological examination, and low number of advanced neoplasms at specific sites has contributed to screening failures [9]. Although noninvasive immunochemical fecal occult blood test (iFOBT) for hemoglobin detection in the stool is available, its sensitivity is comparatively low in detecting early stages of CRC as well as advanced adenomas [10]. Repetitive screening is usually carried out to increase its accuracy, resulting in time-consuming and significant cost implications [11,12]. Concerning the drawbacks as mentioned above, immense interest has been directed towards the discovery of less invasive and easily operated screening systems [13,14].

Previously, marker-based CRC detection methods relied on studying single analyte in a sample. The recent technological advancement in analytical methodologies enables multiple biomarkers to be measured simultaneously [15,16,17]. Unlike traditional enzyme-linked immunosorbent assay (ELISA) or radioimmunoassay, these multiplex technologies have several advantages, including the ability to run several biomarkers from a sample simultaneously, reduced technical error, increased accuracy, and easy translation into clinical practice [18]. Due to this, Human Circulating Cancer Biomarker Multiplex Immunoassay Magnetic Bead Panel 4 was selected. It enabled the binding of multiple color-coded beads to biomarkers present in blood serum, generating various analyte-specific results from a sample. The panel included 10 markers: neural cell adhesion molecule L1 (L1CAM), carbonic anhydrase IX (CA9), mesothelin (MSLN), midkine (MDK), hepsin (HPN), kallikrein 6 (KLK6), transglutaminase 2 (TGM2) aldehyde dehydrogenase 1 family, member A1 (ALDH1A1), epithelial cell adhesion molecule (EpCAM), and cluster of differentiation 44 (CD44). The detailed descriptions and selection rationale of all the markers were as shown in Table 1 below.

## 2. Materials and Methods

### 2.1. Patients and Serum Samples

The database of the Biobank, UKM Medical Molecular Biology Institute (UMBI) was searched for specimen collection. All the specimens in the Biobank were obtained according to the institutional ethical committee approval (UKM PPI/111/8/JEP-2017-583 27 August 2017), and the patients have given informed consent. Sixty CRC patients from Hospital Canselor Tuanku Muhriz were included in the study. All patients had serum samples stored in the Biobank, were Malaysians, and comprised all ages with all stages of CRC. None of the included patients had other types of cancer.

The patients were given a number stage based on their tumor, node, metastasis (TNM) system (T1 or T2, N0, M0: Stage I; T3 or T4, N0, M0: Stage II; any T, N1 or N2, M0: Stage III; any T, any N, M1: Stage IV) or Dukes’ staging (Dukes’ A: Stage I; Dukes’ B: Stage II; Dukes’ C: Stage III; Dukes’ D: Stage IV) as shown in Table 2. They were also categorized based on the presence of distant metastasis, lymph node metastasis, and chemotherapy status.

### 2.2. Luminex Analyser MAGPIX^®^ Multianalyte Profiling of Markers

This study was performed with commercially available MILLIPLEX^®^ MAP Human Circulating Cancer Biomarker Magnetic Bead Panel 4 (Merck KGaA, Darmstadt, Germany) based on the Luminex^®^ xMAP^®^ technology. Serum samples were diluted in assay buffer with a ratio of 1:5. Twenty-five microliters of each diluted sample was added to a mixture of fluorescent-coded magnetic beads precoated with the analyte-specific capture antibody. Binding of the antibodies to the analytes of interest (biomarkers) took placed overnight (16 to 18 h) at 4 °C. Biotinylated detection antibodies were added the next day, followed by an hour incubation at room temperature. Then, Streptavidin–Phycoerythrin conjugate was added into each well to complete the reaction. Each microsphere was identified using Luminex analyzer MAGPIX^®^, and the results were calculated based on fluorescent reporter signals. Mean fluorescence intensities were quantified using the xPonent 4.2 software (Luminex Corporation, Austin, Texas, United States), using a five-parameter logistic curve fitting to derive the analyte concentrations in each sample [72].

### 2.3. Statistical Analysis

The data were first evaluated with the D’Agostino and Pearson omnibus normality tests to determine the normal distribution. For comparisons of variables between two groups, student’s *t*-test or Mann–Whitney U test, depending on normality test results, was performed, whereas one-way analysis of variance (ANOVA) or Kruskal–Wallis test was used to compare groups with three or more variables, followed by post hoc testings (Dunn’s or Tukey’s multiple comparisons test), respectively. Data were expressed as median with standard error and 95% CI. Data were analyzed with GraphPad Prism version 7.0 (GraphPad Software Inc., San Diego, California, United States). On the other hand, the receiver operating characteristic (ROC) curves and area under the curve (AUC) of selected biomarkers were calculated with 95% confidence intervals. A method by Delong et al. was incorporated to compare the ROC curves AUCs and standard error [73]. The cut point (sensitivity and specificity) was chosen based on the Youden index. Logistic regression was carried out to identify the diagnosis accuracy of selected biomarkers. Data were analyzed with MedCalc^®^ version 19.2 (MedCalc Software Ltd., Ostend, Belgium) [74]. All tests were two-sided, and *p*-values of less than 0.05 were observed as statistically significant.

## 3. Results

### 3.1. Clinicopathological Features of Colorectal Cancer Patients

For this study, 60 CRC patients from Hospital Canselor Tuanku Muhriz were chosen. The age of diagnosis ranged from 30 to 89 years old. They were then categorized based on their clinicopathological features, as tabulated in Table 3. They were first divided into two subgroups: primary CRC (36 patients) and mCRC (24 patients). A number stage was also given to each patient based on their TNM system or Dukes’ stage, resulting in 5 Stage I, 7 Stage II, 24 Stage III, and 24 Stage IV CRC patients. Among them, 10 had a history of chemotherapy, and 18 presented with lymph node metastasis.

### 3.2. Association between Primary and Metastatic Tumors

**Figure 1:**
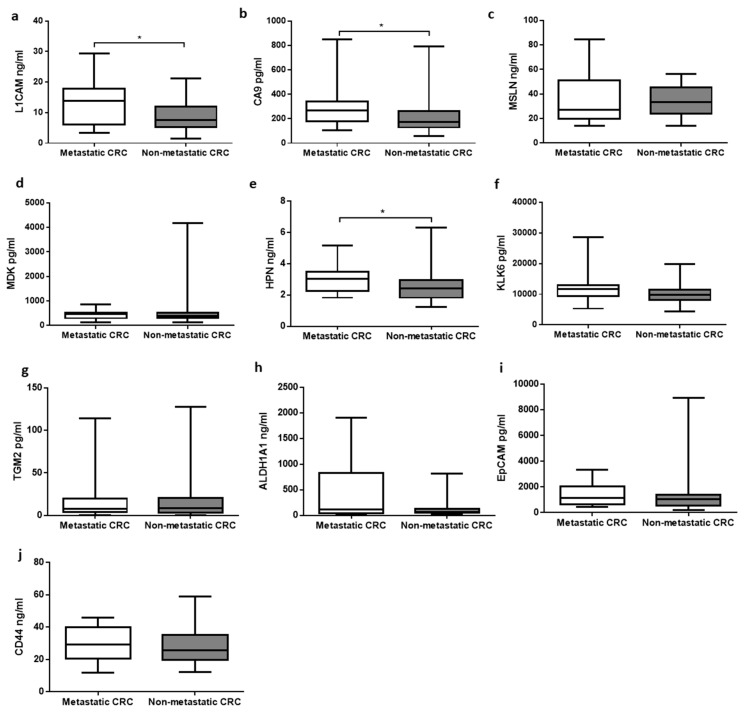
Comparisons of concentrations of biomarkers in serum between primary and mCRC. (**a**) L1CAM (*p* = 0.036); (**b**) CA9 (*p* = 0.014); (**c**) MSLN; (**d**) MDK; (**e**) HPN (*p* = 0.015); (**f**) KLK6; (**g**) TGM2; (**h**) ALDH1A1; (**i**) EpCAM; (**j**) CD44. Results were expressed as median with the lowest (minimum) and largest (maximum) concentrations, and standard error of means. Boxplot with * signified *p* < 0.05 between primary CRC and mCRC.

Serum from a total of 60 patients was collected and assessed using the MAGPIX^®^ system. In order to determine the importance of each marker, concentrations of 10 ten markers (L1CAM, CA9, MSLN, MDK, HPN, KLK6, TGM2, ALGH1A1, EpCAM, and CD44) were compared individually between primary and mCRC. Among them, only 3 of the serum markers showed a significant increase (*p* < 0.05) in mCRC, namely L1CAM (13.77 ± 1.439 ng/mL), CA9 (267.601 ± 35.162 pg/mL), and HPN (3.049 ± 0.172 ng/mL) as compared with primary CRC, respectively (7.619 ± 0.863 ng/mL; 174.567 ± 27.307 pg/mL; 2.427 ± 0.168 ng/mL) (Figure 1).

### 3.3. Relationship between Analytes Concentrations and Clinicopathological Features of CRC

**Figure 2:**
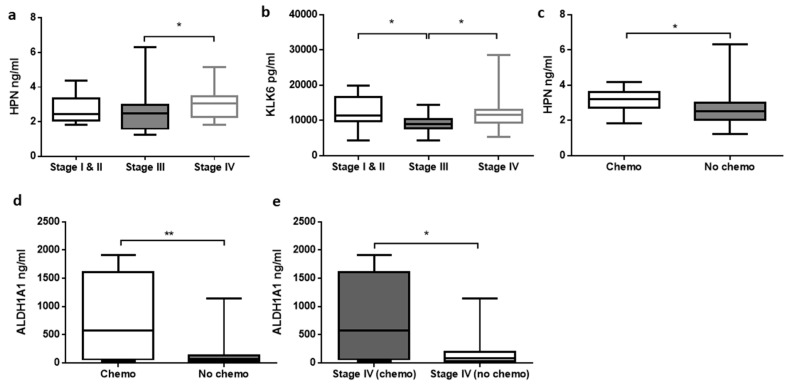
Comparisons of concentrations of biomarkers in serum based on the clinicopathological features: (1) Number staging: (**a)** HPN stage III vs. IV (*p* = 0.034); (**b**) KLK6 stage I & II vs. III (*p* = 0.031) and stage III vs. IV (*p* = 0.023); (2) Chemotherapy status: (**c**) HPN chemotherapy vs. no chemotherapy (*p* = 0.036); (**d**) ALDH1A1 chemotherapy vs. no chemotherapy (*p* = 0.008); (3) Chemotherapy status in stage IV CRC patients: (**e**) ALDH1A1 stage IV chemotherapy vs. no chemotherapy (*p* = 0.048). Results were expressed as median with the lowest (minimum) and largest (maximum) concentrations, and standard error of means. Boxplot with * signified *p* < 0.05 and ** signified *p* < 0.01 among different models.

In an attempt to discriminate CRC on the basis of its clinicopathological features (Figure 2), CRC patients were classified based on their number staging, chemotherapy status, and lymph node metastasis (Table 3). Significant difference (*p* = 0.034) was encountered in HPN concentration between stage III (2.478 ± 0.229 ng/mL) and IV (3.049 ± 0.172 ng/mL) CRC patients. There were significant elevations (*p* < 0.05) of KLK6 in both stage I & II (11,305.650 ± 1325.765 pg/mL) and stage IV (11,606.040 ± 932.493 pg/mL) when compared with stage III CRC patients (8917.764 ± 479.052 pg/mL).

Concentrations of HPN and ALDH1A1 were significantly higher (*p* < 0.05) in CRC patients treated with chemotherapy than in chemotherapy-naive patients. As compared with chemotherapy-naive patients (2.533 ± 0.141 ng/mL), HPN concentration was higher (*p* = 0.036) in CRC patients treated with chemotherapy (3.201 ± 0.225 ng/mL). Similarly, there was an increase in the concentration of ALDH1A1 in chemotherapy-treated patients (581.060 ± 240.426 ng/mL) compared with nontreated ones (66.433 ± 34.389 ng/mL). When only stage IV CRC patients were divided into chemo versus no chemo groups, ALDH1A1 showed significant elevation (*p* = 0.048) in its concentration (581.060 ± 240.426 ng/mL) as compared with the latter (86.016 ± 93.285 ng/mL).

### 3.4. Receiver Operating Characteristic Curve and Logistic Regression Analysis

**Figure 3:**
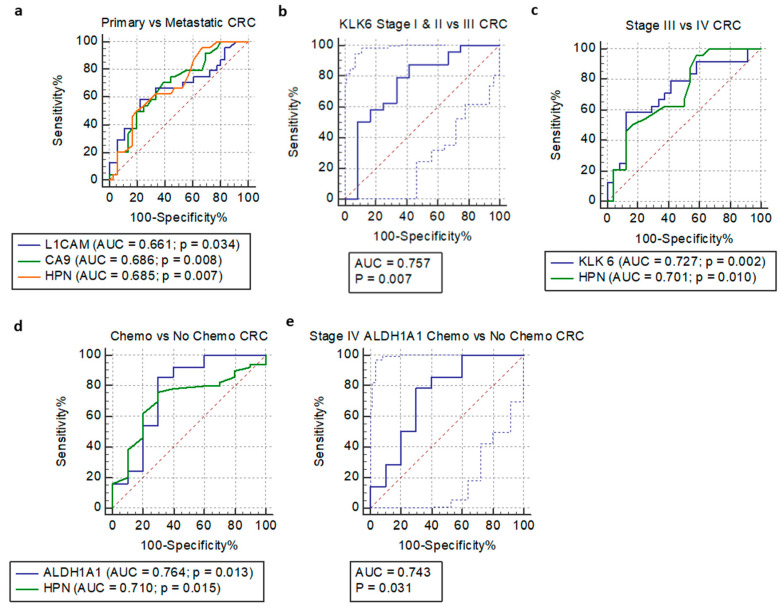
Receiver operating characteristic curves of selected serum biomarkers: (1) Primary versus metastatic CRC: (**a**) L1CAM/CA9/HPN; (2) Number stage: (**b**) KLK6 stage I & II vs. IV; (**c**) KLK6/HPN stage III vs. IV; (3) Chemotherapy versus no chemotherapy: (**d**) ALDH1A1/HPN; and (4) Stage IV chemotherapy versus no chemotherapy: (**e**) ALDH1A1.

In the test accuracy evaluation of selected serum biomarkers, which were significantly different (*p* < 0.05) in the CRC patients, the receiver operating characteristic (ROC) curves were plotted, and the area under the curve (AUC) was calculated (Figure 3, Table 4). The cutoff points were determined based on the Youden index. The highest AUC value associated with primary and metastasized tumors was calculated for CA9 (0.686), followed by HPN (0.685) and L1CAM (0.661).

Among different CRC stages, the highest AUC value of 0.757 was found in a comparison of KLK6 between stage I & II and IV CRC patients (sensitivity = 87.5). Another two models revealed an AUC value of 0.727 with a specificity of 87.50% (KLK6 stage III versus IV) and 0.701 with a specificity of 83.33% (HPN stage III versus IV). However, both models had low sensitivity.

The AUC under ROC curves was also calculated for comparisons of selected biomarkers between chemotherapy and chemotherapy-naïve CRC patients. Out of the two models, both HPN and ALDH1A1 revealed significant AUC values of 0.710 and 0.764, respectively. When stage IV CRC patients were compared based on their chemotherapy status, a significant difference (*p* = 0.031) was only identified in ALDH1A1 with an AUC value of 0.743.

Logistic regression was built to assess the accuracy of the diagnostic markers. Of the selected biomarkers, only five of them are significant (*p* < 0.05): (1) L1CAM primary versus metastatic tumors; (2) KLK6 stage I & II versus III CRC; (3) KLK6 stage III versus IV CRC; (4) ALDH1A1 chemo versus no chemo CRC; and (5) stage IV ALDH1A1 chemo versus no chemo CRC.

### 3.5. Multivariable Logistic Regression Models

Multivariable logistic regression analysis was also carried out to determine if the increment of analytes would further enhance the diagnostic accuracy (Table 4). However, none had significant improvement when two or three biomarkers were compared. Three biomarkers (L1CAM, CA9, and HPN) showed a rise in AUC to 0.719 with a sensitivity of 88.89%, when compared with individual analytes. Comparisons of the regression models in KLK6/HPN between stage III and IV (AUC = 0.727) and HPN/ALDH1A1 chemotherapy versus chemotherapy-naïve CRC patients (AUC = 0.748) did not reveal any significance, although the latter showed the highest specificity of 98.00%.

## 4. Discussion

A serum marker or biomarker is a molecule able to be detected in the serum. It permits identification of a particular disease, in this case, CRC. Thus, marker-based assays have high prognostic and diagnostic values. They are crucial in early CRC detection for treatment selection and prediction of patients’ outcomes [75]. Patient outcomes strongly depend on the tumor stage, metastatic capabilities, localization and presence of distant metastases. Beyond any doubt, early diagnosis is crucial for successful treatment, especially in metastatic CRC. Although several studies were identifying novel serum biomarkers, involving insulin-like growth factor-binding protein 2 (IGFBP-2) [76], heat shock protein 60 (HSP60), and chitinase-3-like protein 1 (CHI3L1) [77], to be strongly correlated with metastasis of CRC, their poor selectivity and sensitivity have rendered the tests unsuccessful due to the high rates of false positives and false negatives. Thus, new biological markers for early diagnosis with higher sensitivity and specificity are urgently needed in clinical practice for better CRC treatment.

Most of the analytes chosen (L1CAM, CA9, MSLN, MDK, HPN, KLK6, TGM2, ALGH1A1, EpCAM, and CD44) have not been studied previously as biomarkers in early detection of CRC. The only scientific evidence involving this panel of markers was published in 2019 by Torres et al. The authors identified CD44, TGM2, and EPCAM as novel plasma markers for endometrial cancer detection [72]. However, a large amount of literature demonstrated the presence of these biomarkers during the progression of CRC (Table 1), suggesting their potential as serum-originated diagnostic markers [19,27,31]. In the present study, a multiplex ELISA-based approach was undertaken due to the advantages over conventional screening methods including (1) high throughput, (2) less sample volume requirements (in microlitres), (3) ability to undergo simultaneous screening of numerous analytes in a sample, (4) ability to use specimens from noninvasive liquid biopsies (serum), (5) ability to evaluate levels of given analyte separately, (6) ability to repeat experimental assay in same conditions, (7) ability to reliably detect analytes across a broad dynamic range of concentrations, (8) increased accuracy, (9) reduced time, labor, and cost, (10) reduced technical errors, and (11) easy translation into clinical practice [18,78,79].

Before conducting the study, the sample size was estimated using G*Power software version 3.1.9.4. We hypothesized fold difference of at least 0.8 between 36 primary CRC and 24 mCRC patients to generate 84.7% power of study [80]. This number provides a balance between providing a precise estimate of accuracy with a wide confidence interval in screening tests and preventing wastage of resources [81]. On the other hand, for the basis estimation of screening tests, sensitivity was predetermined to be at least 50.0% within the null hypothesis, whereas a lower degree of specificity can be tolerated as a screening tool [82].

Based on the results, concentrations of L1CAM, CA9, and HPN were significantly elevated in mCRC as compared with primary CRC samples. The increased concentration of L1CAM and CA9 was in line with several published studies. For example, Kajiwara et al. found out that overexpression of L1CAM was related to CRC tumor budding grade and solid cancer nests [25]. In 2020, Ganesh et al. demonstrated that L1CAM+ cells in human CRC had the metastasis-initiating capacity, and L1CAM was required for orthotropic carcinoma propagation, liver metastatic colonization, and chemoresistance in CRC [22]. On the other hand, CA9, a hypoxia-inducible membrane-tethered protein, was believed to be closely related to carcinogenesis of CRC [83] and linked to poor prognosis of CRC [26]. Overexpression of CA9 in CRC was proven to be correlated with perineural invasion [27], which was a sign of tumor metastasis and invasion as well as an indication of poor outcome in CRC [84]. Another study in 2019 suggested the co-localization of CA9 with phosphorylated ezrin (EZR), activated the hypoxia–autophagy–EZR pathway in tumor-initiating human cells and primary CRC tissues, proving its clinical relevance [85]. In short, the elevation of L1CAM and CA9 concentration was expectable and corresponded to results presented by other authors.

Contrarily, although the concentration of HPN was increased significantly in mCRC when compared with primary CRC, and significant differences were observed among stage IV and chemotherapy-treated CRC patients, there is yet any scientific facts relating its expression with CRC metastasis [35,36]. Nonetheless, the generated data displayed the involvement of HPN in the metastatic progression of CRC. One of the plausible explanations was that overexpression of HPN was associated with matrix degradation (invasion and metastasis initiator), similar to that of prostate cancer [86]. Another assumption was that HPN was strongly associated with pathogenesis and early carcinogenesis of CRC, since it caused disorganization of the basement membrane and promoted primary prostate cancer progression and metastasis to liver, lung, and bone [87]. Curiously, low expression of HPN was associated with poor survival in breast cancer, renal cell carcinoma, and hepatocellular carcinoma [88,89,90]. Still, it was in parallel with the decrease in HPN concentration after chemotherapy treatment.

Accurate preoperative diagnosis is crucial in the management of CRC. If CRC is detected in the early stages, especially when the cancer lesions are localized, patients are likely to have better clinical outcomes [5]. Until today, the majority of the CRC is uncovered just after the appearance of obvious signs and symptoms (usually signifying late stage). Although colonoscopy and iFOBT are the most established CRC screening tests, they are bounded by uptake and adherence [10,91]. Moreover, a rapid noninvasive screening method with high sensitivity and specificity is still unavailable [92]. Due to this, a reasonably accurate procedure in depicting CRC at its earlier stages would reduce its mortality and incidence rates [93]. For that purpose, CRC patients were divided into three groups of different stages (stage I & II, III, and IV), and comparisons were done between the divided groups and selected biomarkers. Although the number of CRC patients for stage I and II were less than of stage III and stage IV, it did not affect the overall statistical power because a weighted mean (each subsample mean was weighted by sample size) was used [94,95], and the power of the study was based on the smallest sample size [96]. Furthermore, since our analysis did not include factorial ANOVA, where sample sizes are confounded in two or more factors, and post hoc tests (Dunn’s test/Tukey) are performed, a possible reduction in statistical power generated was minimized [97]. A significant *p*-value indicated that there was a difference between the groups.

Based on the results, KLK6 expression was significantly elevated in both stages I & II and IV when compared with stage III CRC patients. The former was not surprising since overexpression of KLK6 was often seen in primary CRC tumors and was linked with tumor aggressiveness, enhanced migration, metastatic capabilities, and poor patients’ outcomes [39,40,98]. The latter was undoubtedly in parallel with other previously published studies. For instance, overexpression of KLK6 was related to epithelial–mesenchymal transition during CRC progression [45,99]. In 2019, Chen et al. discovered critical functions of KLK6 enzymes in CRC advancement to late stages via activation of the high mobility group A2 protein [99]. Furthermore, KLK6 expression in CRC correlated significantly with increasing tumor stage and histological grade [100] and was connected with a more advanced Dukes’ stage, liver metastasis, and poor prognosis [37,38,39,40]. Upregulation of KLK6 was also believed to be associated with high depth of tumor invasion, presence of distant metastases, and as an independent prognosticator to predict poor disease-free and overall survival in CRC patients [47]. Conversely, the possible hypothesis for the decrease of KLK6 in stage III CRC patients might be due to its tumor-suppressive [101,102], and immunologic properties since downregulation of KLK6 was associated with the compromise of immune system via regulation of lymphocytes survival and accelerated cancer progression [103].

Based on the analyzed results, the concentration of ALDH1A1 was significantly elevated in chemotherapy-treated and stage IV CRC patients. The possible hypothesis behind this phenomenon could be due to the metastasis progression of CRC itself and not due to chemotherapy since chemotherapy-treated patients all comprised stage IV CRC. To justify, Kahlert et al. revealed that ALDH1A1 expression was not significantly connected with prognosis in CRC and did not predict response to chemotherapy in patients with metastatic diseases [52]. Additionally, ALDH1A1 promoted tumor angiogenesis via retinoic acid/HIF-1α/VEGF signaling [104], while being identified as an indication for poor CRC outcome [53,54].

Initially, a priori analysis was done to determine the power of study of at least 80% among the included 60 CRC patients, but later we found out that even with our careful study design choices to minimize bias, samples exhibited large variations within each biomarker. This might be due to the unevenly distributed range and overlapping of marker concentrations, coupled with person-to-person variation from the samples [105]. Heterogeneity within clinical samples is crucial in producing data with less variation and more precision [106]. Increasing sample size might not be the solution and will magnify biases if selected samples show similar data distribution patterns, which do not reflect the total CRC population [107,108]. Concisely, heterogeneity within samples was preferred over the large sample sizes.

One of the research questions in this research was to confirm whether the increment of two or three serum biomarkers would enhance diagnostic accuracy. Unfortunately, none of the combinations of selected biomarkers showed significant improvement. Even if there was an improvement in either sensitivity or specificity, the remaining had decreased value, or there was no improvement at all for both. We predict that this could be due to the presence of outliers and the limited population of CRC patients. To train a reliable model, the inclusion of more data distribution patterns of CRC is needed.

Our study is not without limitations. Even though the sample size is 60, this preliminary study still has sufficient power to detect several significantly expressed circulating markers that hold the potential for future exploration. Additionally, since tumor tissue-derived proteins from the serum are likely to be low and diluted, especially during early stages of cancer, expression analysis of tumor tissue, for instance, immunohistochemistry and quantitative PCR, could be done to identify markers that are both tissue-specific and upregulated in CRC.

## 5. Conclusions

In conclusion, this study reported several biomarkers from serum (L1CAM, CA9, KLK6, HPN, and ALDH1A1) that could act as a potential noninvasive screening tool for CRC, but further additional testings are needed.

## Figures and Tables

**Table 1:** Selection rationale of the 10 markers from the Human Circulating Cancer Biomarker Multiplex Immunoassay Magnetic Bead Panel 4.

Marker	Role in Colorectal Cancer	Citation
L1CAM	• A member of the immunoglobulin-like cell adhesion molecule family that is shown to be associated with a worse prognosis of CRC.	[19,20,21]
	• A major driver for tumor formation and metastasis initiation capability in CRC.	[22,23]
	• L1CAM contributes to peritoneal dissemination in CRC.	[24]
	• The regulation of L1CAM is strongly correlated with morphologic features at the invasive front in CRC.	[25]
CA9	• A transmembrane glycoprotein involved in cell proliferation, angiogenesis, and a marker for hypoxia and acidosis, which is linked to poor prognosis in CRC.	[26]
	• CA9 correlates with perineural invasion in CRC.	[27]
MSLN	• A cell surface membrane-bound glycoprotein which is highly expressed in solid CRC tumors (40–45%).	[28,29]
	• MSLN acts as a prognostic marker for stage ΙΙ/ΙΙΙ CRC.	[30]
MDK	• A heparin-binding growth factor, which induces neo-lymphangiogenesis and exhibits anti-apoptotic, migration-promoting, and angiogenic properties.	[31]
	• Overexpression of MDK in the blood of CRC patients indicates a worse prognosis. MDK generally increases with increasing severity of cancer.	[32,33,34]
	• MDK adds value to multi-marker CRC biomarker panels.	[34]
HPN	• A cell-surface type II transmembrane serine protease with genetic alteration in colon carcinoma (1.2%).	[35,36]
KLK6	• A trypsin-like serine protease that is upregulated in tissues and sera from patients with malignant colon tumors compared with normal tissues.	[37,38,39,40]
	• Upregulation of KLK6 protein is associated with a more advanced Dukes’ stage, serosal invasion, liver metastasis, and unfavorable predictor of overall survival among CRC patients.	[41,42,43,44]
	• KLK6 regulates epithelial–mesenchymal transition in CRC progression via the TGF-β- (transforming growth factor beta-) signaling pathway.	[45]
	• *KLK6* mRNA overexpression is associated with high depth of tumor invasion, presence of distant metastases, and tumor, node, metastasis (TNM) stage of patients.	[44,46,47]
	• *KLK6* mRNA expression is shown to predict poor disease-free and overall survival independently of patient gender, age, tumor size, location, histological subtype, grade, venous invasion, lymphatic invasion, TNM stage, radiotherapy, and chemotherapy treatment.	[47]
TGM2	• A well-known apoptosis attenuator. TGM2 is transcriptionally activated by protein C-eta-1 (ETS1) and inhibits apoptosis, angiogenesis. TGM activates Wnt/β-catenin signaling, resulting in chemotherapy-related stress.	[48,49]
	• Higher expression of TGM2 indicates a poorer overall survival rate (independent prognostic marker).	[50]
	• TGM2 affects the metastatic potential, self-renewal, and stemness of CRC stem cells by regulating epithelial–mesenchymal transition- and stemness-related proteins.	[51]
ALDH1A1	• A cancer stem cell marker, where its nuclear expression is associated with shortened overall survival in CRC patients.	[52]
	• Overexpression of ALDH1A1 in CRC is associated with the presence of lymph node metastases and poor prognosis.	[53,54]
	• ALDH1A1 expression is associated with poor differentiation, “right-sidedness”, and poor survival in human CRC.	[55]
EpCAM	• A transmembrane glycoprotein cell adhesion molecule highly expressed on the surface of epithelium-originated tumor cells, including CRC. High expression of EpCAM is linked with an aggressive tumor phenotype in primary stages of CRC.	[56,57,58,59]
	• Loss or reduced expression of EpCAM during disease progression stage is linked with aggressive tumor phenotype AM, tumor differentiation, tumor staging, vascular invasion, depth of tumor invasion, lymph node metastasis, distant metastasis, and tumor budding in CRC.	[60,61,62,63,64,65]
	• EpCAM-based assay (the CellSearch^®^ System) is the only FDA-approved test for enrichment and detection of circulating tumor cells of cancers, including CRC.	[66]
CD44	• A common CRC stem cell marker and is associated with tumor initiation, tumor progression, tumor growth, invasion, and metastasis.	[67,68,69]
	• Overexpression of CD44 in colon tissue is associated with cancer progression, aggressiveness in stage I and III sporadic CRC, poor differentiation, lymph node metastasis, and distant metastasis.	[70]
	• Low alteration frequency of *CD44* found in mRNA is linked with the prediction of prognosis in CRC.	[71]

ALDH1A1: aldehyde dehydrogenase 1 family, member A1, CA9: carbonic anhydrase IX, CD4: cluster of differentiation 44, CRC: colorectal cancer, EpCAM: epithelial cell adhesion molecule, FDA: Food and Drug Administration, HPN: hepsin, KLK6: kallikrein 6, L1CAM: neural cell adhesion molecule L1, MDK: midkine, mRNA: messenger ribonucleic acid, MSLN: mesothelin, TGM2: transglutaminase 2.

**Table 2:** Assignment of number stage.

Number Stage	TNM System and Duke Staging
Stage I	T1 or T2, N0, M0 or Dukes’ A
Stage II	T3 or T4, N0, M0 or Dukes’ B
Stage III	Any T, N1 or N2, M0 or Dukes’ C
Stage IV	Any T, any N, M1 or Dukes’ D

**Table 3:** Summary of characteristics of the included CRC patients.

Characteristics		Number of Patients
Gender	Female	20
	Male	40
Distant metastatic CRC	Absent	36
	Present	24
Number staging	Stage I	5
	Stage II	7
	Stage III	24
	Stage IV	24
Chemotherapy	Absent	50
	Present	10 (all Stage IV)
Lymph node metastasis	Absent	42
	Present	18

**Table 4:** Receiver operating characteristic (ROC) curves results and logistic regression models.

Model	Sensitivity (%)	Specificity (%)	ROC AUC	95% CI	ROC *p*-Value	SE	dAUC	*p*-value
**Primary vs. metastatic CRC**								
L1CAM	58.330 *	77.780 *	0.661	0.527–0.778 **^a^**	0.034	0.076 **^b^**	/	0.034
CA9	70.830 *	61.110 *	0.686	0.554–0.800 **^a^**	0.008	0.070 **^b^**	/	0.345
HPN	50.000 *	80.560 *	0.685	0.552–0.799 **^a^**	0.007	0.069 **^b^**	/	0.345
L1CAM/CA9/HPN	88.890	50.000	0.719	0.588–0.827	/	0.071	/	/
**Number stage**								
KLK6 stage I & II vs. III	87.500 *	58.330 *	0.757	0.586–0.884 **^a^**	0.007	0.095 **^b^**	/	0.023
KLK6 stage III vs. IV	58.330 *	87.500 *	0.727	0.580–0.846 **^a^**	0.002	0.075 **^b^**	/	0.023
HPN stage III vs. IV	50.000 *	83.330 *	0.701	0.551–0.824 **^a^**	0.010	0.078 **^b^**	/	0.111
KLK6/HPN stage III vs. IV	66.670	62.500	0.727	0.580–0.846	0.868	0.074	0.013	/
**Chemo versus no chemo**								
HPN	76.000 *	70.000 *	0.710	0.578–0.820 **^a^**	0.015	0.086 **^b^**	/	0.858
ALDH1A1	86.000 *	70.000 *	0.764	0.637–0.864 **^a^**	0.013	0.106 **^b^**	/	0.003
HPN/ALDH1A1	40.000	98.000	0.748	0.619–0.851	0.466	0.109	0.054	/
**Stage IV chemo versus no chemo**								
ALDH1A1	78.570 *	70.000 *	0.743	0.525–0.898 **^a^**	0.031	0.110 **^b^**	/	0.039

* The sensitivity and specificity (cut point) were determined based on the Youden index; **^a^** Binomial exact; **^b^** Delong et al., 1988 [73]; **^c^** Logistic regression, ALDH1A1: aldehyde dehydrogenase 1 family, member A1, AUC: area under the curve, CA9: carbonic anhydrase IX, CRC: colorectal cancer, chemo: chemotherapy, CRC: colorectal cancer, dAUC: difference in AUC, HPN: hepsin, KLK6: kallikrein 6, L1CAM: neural cell adhesion molecule L1, ROC: receiver operating characteristic, SE: standard error.

## Abbreviations

ALDH1A1: aldehyde dehydrogenase 1 family, member A1, ANOVA: analysis of variance, AUC: area under curve, CA9: carbonic anhydrase IX, CD4: cluster of differentiation 44, chemo: chemotherapy, CRC: colorectal cancer, dAUC: difference in AUC, EpCAM: epithelial cell adhesion molecule, HPN: hepsin, iFOBT: immunochemical fecal occult blood test, KLK6: kallikrein 6, L1CAM: neural cell adhesion molecule L1, mCRC: metastatic CRC, MDK: midkine, mRNA: messenger ribonucleic acid, MSLN: mesothelin, ROC: receiver operating characteristic, SE: standard error, TGM2: transglutaminase 2.
